# Perceptions and Barriers to Administering Vasopressors in the Prehospital Setting

**DOI:** 10.7759/cureus.29614

**Published:** 2022-09-26

**Authors:** Eric Quinn, John Su, Lorraine Fei, Joseph Liu, Matt Friedman, David Lobel, Sarah Kabiriti, Antonios Likourezos, Sergey Motov, David Eng

**Affiliations:** 1 Emergency Medicine, Maimonides Medical Center, Brooklyn, USA

**Keywords:** paramedic, fluid-refractory shock, vasopressor, prehospital emergency medicine, epinephrine, hypotension, norepinephrine

## Abstract

Introduction

Vasopressor administration is a critical medical intervention for patients with hypotension in undifferentiated shock states. Over the years, prehospital care has advanced with protocols and training that allow paramedics in the field to administer a variety of vasopressors. The primary objective of this investigation was to evaluate vasopressor experience among paramedics with regard to preference as well as the barriers to its preparation and administration.

Methods

A cross-sectional survey of vasopressor use by nationally certified paramedics (NRPs) was performed. A 20-item questionnaire was constructed to capture the prehospital perceptions and barriers of dopamine infusion, norepinephrine infusion, and IV bolus “push-dose” epinephrine (PD-E). Data collection was carried out from June to September 2021.

Results

A total of 44 responses were obtained (response rate = 44%). All participants had experience using vasopressors and understood their medical indications. Overall, PD-E was the most common vasopressor used in the prehospital setting, and participants felt equally confident in “using” and “preparing” it. Participants felt less confident with “using” and “preparing” vasopressors that required channel setup and maintaining a flow rate. Younger paramedics with less than five years of experience were more eager to use norepinephrine if trucks were stocked with pre-mixed norepinephrine rather than the current formulation that required compounding.

Conclusion

This study provided preliminary data that evaluated perceptions of vasopressor use in the prehospital setting among paramedics in a large urban environment. Preference and barriers to its preparation and administration were surveyed. Further research is needed to identify the interventions to reduce barriers and allow paramedics to be less limited by logistical considerations when choosing vasopressors in the prehospital setting.

## Introduction

Treatment of hypotension is a crucial component of patient care, both in the prehospital and in-hospital settings, to prevent morbidity and mortality [1-5]. A review of the available literature suggests that 2% of patients in the prehospital setting present with hypotension with an in-hospital mortality rate between 33% and 52% [6]. Vasopressor administration is a critical medical intervention for patients with hypotension and shock. However, successful management of the hypotensive patient is difficult in the uncontrolled prehospital environment. Transportation decisions and other concomitant disease processes that need to be addressed in the resource-limited setting make the prehospital treatment of shock with vasopressors challenging.

Over the years, prehospital care has advanced with protocols and training that allow paramedics in the field to administer a variety of vasopressors, including dopamine, epinephrine, and norepinephrine. Vasopressor use was long believed to require central venous access, limiting prehospital usage, along with the complexity of establishing weight-based infusions and the unavailability of infusion pumps. However, recent literature now supports the use of intravenous vasopressors via peripheral access in terms of safety and efficacy [7-9].

Vasopressors are not utilized often, but when indicated are used on the most critical patients. In 2019, our local regional-based Emergency Medical Services (EMS) agency in Brooklyn, New York (NY), which is dispatched to around 30,000 calls annually, administered vasopressors in the prehospital setting only 30 times (0.1%). Within the prehospital literature, the evidence is limited by the paramedics’ experience in utilizing the variety of vasopressors in the field. The primary objective of this investigation was to evaluate vasopressor experience among paramedics with regard to preference as well as the barriers to its preparation and administration.

## Materials and methods

Study design and population

A cross-sectional survey of vasopressor use by nationally certified paramedics (NRPs) was performed. The population of interest for this study were advanced life support (ALS) paramedics at Maimonides Medical Center (MMC). There were a total of 100 ALS full- and part-time paramedics, all with varying years of experience. Basic life support (BLS) personnel were excluded from this study. The study was reviewed and exempted by the MMC Institutional Review Board.

Study setting

The study was conducted by the Prehospital Division in the Department of Emergency Medicine at MMC, a single community site in Brooklyn, NY. MMC features 14 ambulances in New York City (NYC), 911 EMS system, and three critical care interfacility ambulances. MMC EMS system responds to approximately 30,000 911 calls annually and has two physician response vehicles in service. Epinephrine requiring dilution, pre-mixed dopamine, and norepinephrine requiring reconstitution are available in the system. The medications can be administered intravenously (IV) or intraosseously. Protocols, where vasopressors can be administered, include "Shock," "Sepsis," and "Brady-dysrhythmia." Dopamine infusion can be given via flow rate meter or channel at 5 mcg/kg/min with a maximum rate of 20 mcg/kg/min. Norepinephrine infusion can be given via flow rate meter or channel at 2 mcg/min with a maximum rate of 20 mcg/min. Lastly, 10 mcg of IV bolus “push-dose” epinephrine (PD-E) over one minute can be given. The three vasopressors can be titrated or repeated every 3-5 minutes.

Study instrument and variable description

**Figure 1:**
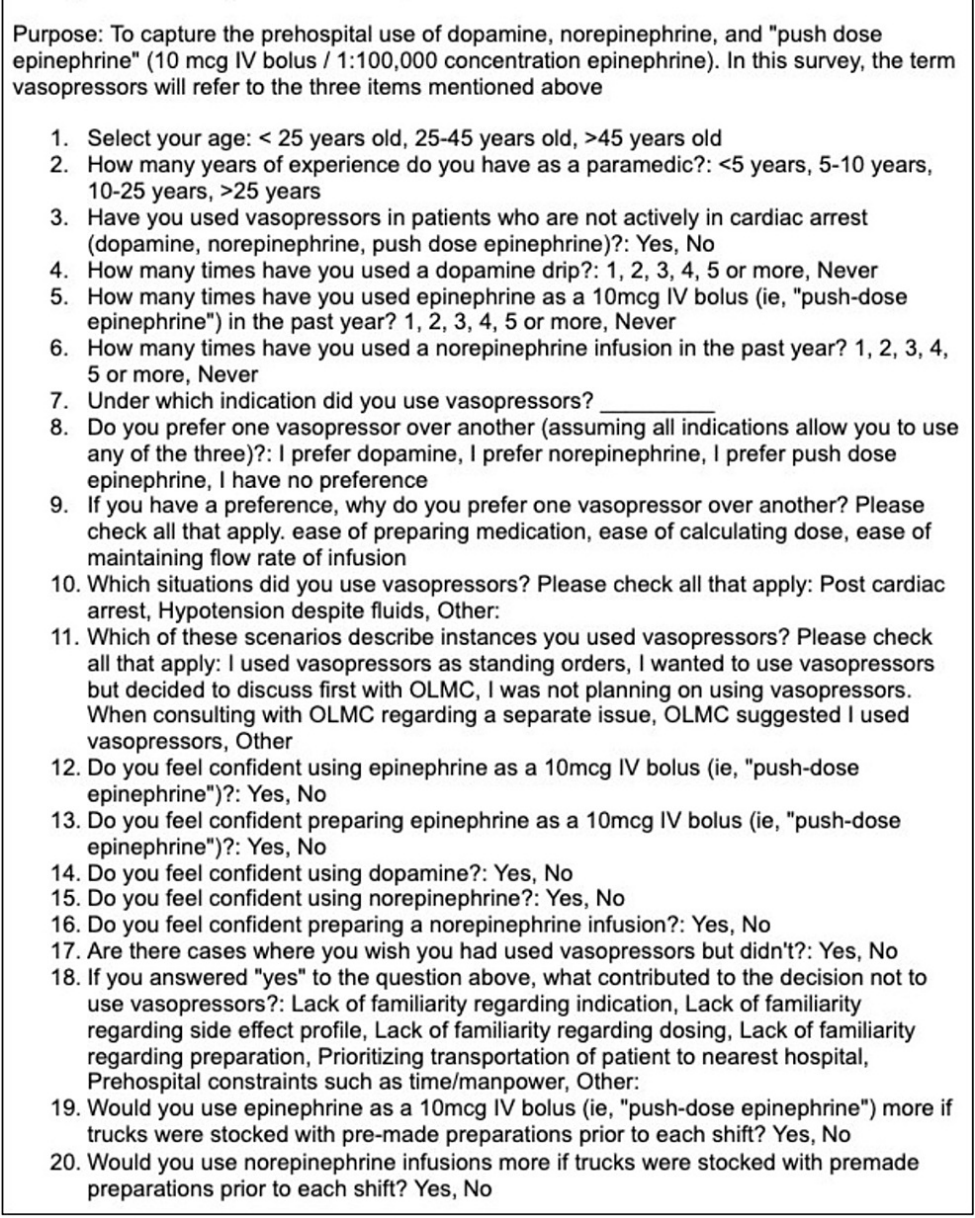
Survey on the prehospital use of vasopressors

A 20-item questionnaire (Figure 1) was constructed by experienced item writers in the prehospital division in the department of emergency medicine to capture the prehospital use of dopamine infusion, norepinephrine infusion, and PD-E. Before administering the questionnaire electronically, commonly used terms were thoroughly defined in the email to ensure all participants understood the items being evaluated.

Participants were first asked to indicate their age (<25, 25-45, >45 years), their years of experience as a paramedic (<5, 5-10, 10-25, >25), and if they had ever administered vasopressors. If participants answered “Yes,” they would then be asked to answer under which indications they administered vasopressors, which will be followed by a series of questions about how often they either used a dopamine infusion, norepinephrine infusion, or PD-E in the past year. Next, participants were asked if they had a strong preference for any one of the three vasopressors, regardless of the vasopressors they used in the prehospital setting.

For the given vasopressor of choice, participants were questioned about why they selected the medication in regard to (1) ease of preparing the medication, (2) ease of calculating dose, and (3) ease of maintaining the flow rate of infusion. The participants were then asked to rate their level of confidence in “preparing” and “using” each of the three vasopressor agents in the following section of the survey.

The term “preparing” was defined to participants with familiarity in reconstituting the medication to a specific concentration as indicated. The term “using” was defined to participants with familiarity in titrating the medication to the clinical effect and maintaining the flow rate of infusion. Participants were also asked if they had any encounters with patients for whom they wished they had used vasopressors but did not. Lastly, given the opportunity for more pre-made preparations of PD-E vs norepinephrine infusion, participants were asked which they would prefer. Currently, in the formulary, both medications must be prepared by the medics.

Data collection and analysis

Data collection was carried out from June to September 2021. All ALS crew members with National EMS Certification were sent an email with a unique survey link to complete the electronic questionnaire. The email included an explanation regarding the goals of the study and every participant’s voluntary right to complete the survey with no punitive consequences or monetary incentives. No interventions were performed in this survey. Data was collected using RedCap (Vanderbilt University, Nashville, TN USA), a secure web application for building and managing online surveys and databases. No identifying information was collected by the survey software.

For data analysis, inclusion criteria consisted of ALS crew members as their primary role. Only completed surveys were used for analysis and were statistically calculated using IBM SPSS (IBM Corp, Armonk, NY). The data between the comparison groups were analyzed using a chi-square test.

## Results

A total of 44 responses were obtained (response rate = 44%) of which 100% met inclusion criteria and constituted the study cohort. Eighty-six percent (n = 38) of participants were between the ages of 25 and 45 years. Given the years of paramedic experience, 47% (n = 21) had less than five years, 27% (n = 12) had between five and 10 years, and 23% (n = 10) had between 10 and 25 years (Table 1). All participants (n = 44) have used at least one of the three vasopressors. Thirty-seven percent of participants (n = 16) have used all three vasopressors. Within the past year, 36% (n = 16) used dopamine infusion, 23% (n = 10) used norepinephrine infusion, and 41% (n = 18) used PD-E (Table 2).

**Table 1:** Demographic profile of respondents to the survey (n = 44)

	Frequency (%)
Age (years)	
<25	3 (6.8%)
25 to 45	38 (86.4%)
>45	3 (6.8%)
Years of experience	
<5	21 (47.7%)
5 to 10	12 (27.3%)
10 to 25	10 (22.7%)
>25	1 (2.3%)

**Table 2:** Participants’ vasopressor use in the past year (n = 44) PD-E: “Push-dose” epinephrine.

	Frequency (%)
Vasopressor	
Dopamine	16 (36%)
PD-E	18 (41%)
Norepinephrine	10 (23%)

The most common indications for vasopressor use were in hypotension (86%, n = 38) and post-cardiac arrest (75%, n = 33). The majority (93%, n = 41) used vasopressors as standing orders. The majority (91%, n = 40) did not require help from Online Medical Control (OLMC) prior to carrying out orders for vasopressors.

Overall, the majority (52%, n = 23) preferred PD-E, while 23% (n = 10) preferred a norepinephrine infusion, and 11% (n = 5) preferred a dopamine infusion. Fourteen percent (n = 6) marked that they did not have a preference (Table 3). The majority picked their preferred vasopressor of choice based primarily on the ease of preparing the medication (59%, n = 26) and ease of calculating the dose (52%, n = 23). Sixty-six percent (n = 29) of participants did not prefer using vasopressors that required them to maintain an infusion flow rate. This is in line with the study group’s overall preference for PD-E. Participants who preferred norepinephrine were overwhelmingly those with less than five years of paramedic experience (43%, n = 9), while only one medic with greater than five years of paramedic experience (4%, n = 1) strongly preferred norepinephrine (p = .026). However, there were no differences between the years of paramedic experience with regard to preference for dopamine infusion and PD-E use (Table 4).

**Table 3:** Participants’ overall vasopressor preference (n = 44) PD-E: “Push-dose” epinephrine.

	Frequency (%)
Vasopressor	
Dopamine	5 (11%)
PD-E	23 (52%)
Norepinephrine	10 (23%)
No preference	6 (14%)

**Table 4:** Participants’ vasopressor preference breakdown by years of experience (n = 44) PD-E: “Push-dose” epinephrine.

	Frequency (%)		P-value
	< 5 years (n = 21)	>5 years (n = 23)	
Vasopressor			
Dopamine	0	3 (13%)	.076
PD-E	12 (57%)	13 (57%)	.214
Norepinephrine	9 (43%)	1 (4%)	.026
No preference	0	6 (26%)	.063

In regard to the participants’ confidence involving each vasopressor, the overwhelming majority felt confident in “using” (96%, n = 42) and “preparing” (98%, n = 43) PD-E (Table 5). Participants felt less confident with “using” (82%, n = 36) and “preparing” (80%, n = 35) dopamine infusion. Interestingly, even less felt confident in “using” (77%, n = 33) and “preparing” (77%, n = 33) norepinephrine infusion. There was no statistical difference in the years of paramedic experience and vasopressor preference with regard to “using” and “preparing” each of the vasopressors.

**Table 5:** Participants’ confidence in “preparing” and “using” vasopressor agents (n = 44) PD-E: “Push-dose” epinephrine.

Vasopressor	Frequency (%)
Dopamine	
“Using”	36 (82%)
“Preparing”	35 (80%)
PD-E	
“Using”	42 (96%)
“Preparing”	43 (98%)
Norepinephrine	
“Using”	36 (82%)
“Preparing”	33 (77%)

Lastly, the majority (70%, n = 31) preferred norepinephrine infusion when participants were given the option to have more pre-made preparations stocked in ambulances for either PD-E or norepinephrine infusion (Table 6). Dopamine already comes pre-made in the formulary. Interestingly, there was a significant difference (p = .044) in the paramedics with less than five years of experience who dramatically preferred the pre-made norepinephrine infusion (91%, n = 19) compared to paramedics with greater than five years of experience (52%, n = 12). This was also true vice versa where there was a significant difference (p = .036) for paramedics with less than five years of experience (48%, n = 11) who dramatically preferred the pre-made PD-E compared to paramedics with greater than five years of experience (9%, n = 2).

**Table 6:** Vasopressor preference of pre-made PD-E versus norepinephrine broken down by years of experience PD-E: “Push-dose” epinephrine.

	Frequency (%)		P-value
	<5 years (n = 21)	>5 years (n = 23)	
Vasopressor			
PD-E	2 (9%)	11 (48%)	.036
Norepinephrine	19 (91%)	12 (52%)	.044

## Discussion

This study provided preliminary data that evaluated perceptions on vasopressor use in the prehospital setting among ALS paramedic providers in a large urban environment with regard to preference as well as the barriers to its preparation and administration. Historically, paramedics have been trained to routinely administer IV fluid in the resuscitation of the hypotensive patient, which may have a limited therapeutic effect. As paramedics frequently encounter unstable patients, pharmacologic interventions that have traditionally been reserved for the in-hospital setting need continued further adoption in the prehospital setting. In this study, it was encouraging to know that all participants had experience using vasopressors and understood the appropriate medical indications prior to usage.

Overwhelmingly, PD-E was the most commonly used agent in the prehospital setting and was the most preferred vasopressor of choice, based chiefly on the ease of preparing the medication and calculating the dose. This makes practical sense since there is no need to maintain an infusion flow rate, which is required for dopamine and norepinephrine. One paramedic mentioned using PD-E avoids the risk of an infusion line being entangled during transport. Preparation and administration of a vasopressor infusion are time-consuming; therefore, many arguments have been made that PD-E boluses are a good bridge to definitive treatment while on the scene [9-11]. The vast majority of the participants, regardless of vasopressor preference or years of paramedic experience, felt confident with “preparing” and “using” PD-E.

Despite the participants' strong preference for PD-E, several studies highlight norepinephrine as the first-line treatment for hypotension, given its better safety profile with respect to dopamine and epinephrine [12-14]. There is an interesting relationship among the participants with less than five years of paramedic experience who would prefer to use norepinephrine more if it was pre-made and readily stocked in ambulances, despite being less confident in “preparing” and “using” norepinephrine. This is contrary to participants with greater than five years of paramedic experience who still prefer more routine use of dopamine and PD-E. It perhaps highlights the current training younger paramedics are receiving and the willingness they have to adopt newer practices compared to other paramedics. Furthermore, the lower rates of confidence with “preparing” and “using” vasopressors that require infusion pumps continue to raise a frequent concern among critics on whether paramedics can adequately administer these agents without medication errors or significant delays. Similar concerns are brought up with emergency physicians attempting to mix and administer bolus medications in the emergency department [8,15]. The above discussion highlights the gaps that are still needed to facilitate the ease of using these medications.

This study was limited by its cross-sectional nature, the small sample size, and the fact that patients were treated in a large urban setting with easy access to multiple hospitals within minutes. Larger sample sizes involving multiple surrounding regional hospitals and nonurban areas are needed to further investigate the validity of these findings.

## Conclusions

This study provided preliminary data that evaluated the perceptions of vasopressor use in the prehospital setting among paramedic providers in a large urban environment with regard to preference as well as the barriers to its preparation and administration. Overall, the majority preferred PD-E and felt confident in “using” and “preparing” PD-E. Although norepinephrine is considered the first-line treatment for hypotension, many felt less confident with “using” and “preparing” medications that require setting up a channel and maintaining a flow rate. Significant results showed that younger paramedics with less than five years of experience were more eager to use norepinephrine if emergency vehicles were readily stocked with them. Further research is needed to identify the interventions to reduce barriers and allow paramedics to be less limited by logistical considerations when choosing vasopressors in the prehospital setting.
